# An epidemiological investigation into the association between biomarkers and growth performance in nursery pigs

**DOI:** 10.1186/1746-6148-9-247

**Published:** 2013-12-06

**Authors:** Mackenzie J Slifierz, Robert Friendship, Cornelius FM de Lange, Marko Rudar, Abdolvahab Farzan

**Affiliations:** 1 Department of Population Medicine, Ontario Veterinary College, University of Guelph, 50 Stone Road, N1G 2 W1 Guelph, Ontario, Canada; 2 Department of Animal and Poultry Science, University of Guelph, 50 Stone Road, N1G 2 W1 Guelph, Ontario, Canada

**Keywords:** Biomarker, Pig, ADG

## Abstract

**Background:**

Biomarkers are useful tools in research and clinical practice where they are often used to detect and monitor differences in the physiological state of an animal. The proteins IGF-1, IGFBP-3, GHR, CRP, SAA, Hp, IFN-α, IFN-γ, TNF-α, IL-1β, IL-6, IL-10, and IL-18 have been proposed as potential biomarkers for monitoring growth in livestock. The objective of this study was to determine whether hepatic gene expression of these proposed biomarkers is associated with growth performance in nursery pigs. Herd information and growth parameters were collected for 168 piglets from 8 commercial farms in southern Ontario. From these pigs, a subset of liver tissue samples (n = 74) was used for gene expression analysis of the proposed biomarkers. Multivariable linear regression methods were used to determine whether genetic expression of the proposed biomarkers was associated with growth performance in the nursery.

**Results:**

Modelling the herd information and individual piglet traits in relation to growth performance revealed that the weight at weaning and the age at weaning are significantly associated with nursery performance. Average daily gain (ADG) was significantly associated with hepatic IGFBP-3 and GHR expression in the liver (*P* < 0.05), and tended to be associated with hepatic IGF-1 expression (*P* = 0.071). Similarly, 9-week body weight was significantly associated with hepatic expression of IGFBP-3 and GHR expression (*P* < 0.05), and tended to be associated with hepatic expression of IGF-1 (*P* = 0.055).

**Conclusion:**

The age and weight at which pigs are weaned is an important determinant for nursery performance. Hepatic gene expression of IGF-1, IGFBP-3, and GHR can be useful biomarkers for monitoring growth performance in nursery pigs.

## Background

The insulin-like growth factor (IGF) system is important for development and tissue growth in livestock species. Insulin-like growth factor-1 (IGF-1) is primarily synthesized by the liver and is crucial for stimulating cellular proliferation and differentiation in brain, muscle, and bone tissue [1]. The expression of IGF-1 is mediated by growth hormone (GH), a peptide hormone released by the anterior pituitary gland [2]. The binding of GH to growth hormone receptors (GHR) in hepatic tissue induces expression of IGF-1 [3]. IGF-1 expression is associated with increased body weight as demonstrated by transgenic over-expression of IGF-1 in mice [4]. The transportation and bioavailability of IGF-1 is facilitated by IGF binding proteins (IGFBPs) which circulate in the blood [5]. The most abundant is IGFBP-3 which binds most circulating IGF-1 [6]. Experiments using a transgenic mouse model have demonstrated that over-expression of IGFBP-3 is associated with growth impediment [7]. In pigs, previous research suggests that serum IGF-1 is positively associated with average daily gain (ADG) and serum IGFBP-3 is negatively associated with ADG [8]. Additional studies have demonstrated that the IGF system is important for postnatal maturation and the development of desirable carcass traits [9-11]. Although these findings demonstrate that the IGF system plays an important role in development, it is not clear to what extent differences in gene expression affect growth performance in pigs, and whether these genes can be used as biomarkers to monitor growth performance across different commercial farms.

There are previous studies which also suggest that growth performance in pigs may be associated with changes in serum concentrations of acute-phase proteins (APP), including C-reactive protein (CRP), haptoglobin (Hp), and serum amyloid A (SAA) [12-15]. It has been proposed that Hp may be a suitable biomarker for monitoring production performance on commercial swine farms [15]. Serum levels of certain cytokines, including interleukin-1β (IL-1β), interleukin-6 (IL-6), interleukin-8 (IL-8), interleukin-18 (IL-18), and tumor necrosis factor-α (TNF-α), have also been associated with body weight in horses and in humans [16-20]. However, the usefulness of these APPs and cytokines as biomarkers for monitoring the growth performance of pigs on commercial swine farms has not been established. The objective of this study was to investigate the association between the hepatic gene expression of potential biomarkers, including IGF-1, IGFBP-3, GHR, CRP, SAA, Hp, IFN-α, IFN-γ, TNF-α, IL-1β, IL-6, IL-10, IL-18, and growth performance in nursery pigs across diverse commercial farm conditions. A secondary objective of this study was to determine extraneous factors which may influence growth performance in the nursery as such factors are critical for assessing the association between potential biomarkers and nursery performance.

## Methods

### Experimental design and sampling

The use of animals in this study was approved by the Animal Care Committee at the University of Guelph. Eight swine farms in southern Ontario were recruited to participate in a collaborative field study. On each farm, seven sows were randomly enrolled in the study and three piglets (small, medium, and large size) were selected from each sow at weaning and ear tagged. In total 168 piglets (21 piglets per farm) were included. All male piglets recruited in this study were castrated. A questionnaire was administered and information about the enrolled sows (farrowing date, parity, litter size, numbers of live born, stillborn), farm management (pig flow, number of gilts, sows, nursery pigs, and grower-finishers, and type of farrowing room floor), health status (recent diseases, mortality in different stage of production), vaccination, and in-feed drug use was collected. Piglets were weighed at weaning and once again at 5 weeks post-weaning. Fecal and blood samples were collected at weaning, 2 weeks post-weaning, and 5 weeks post-weaning.

At 5 weeks post-weaning the piglets were transported to the University of Guelph where they were euthanized. Tissue samples collected immediately from the liver, intestinal lymph node, ileum, and spleen were placed immediately into RNAlater and allowed to sit at 4°C for 24 h before being stored at -80°C. Samples were stored at -80°C for no longer than 4 weeks before being processed for gene expression analysis. Separate portions of the ileum and colon were collected and nasal swabs, blood, and fecal samples were collected from each pig prior to euthanasia.

### Calculating growth performance

Growth performance was determined by assessing the post-weaning ADG and the 9 week body weight (9-wk BW) of each nursery piglet. ADG was calculated as the difference between the weaning weight and the 5-wk post-weaning weight, divided by the number of days between each weighing. Body weights were standardized to 9 weeks of age as there was considerable variation in piglet age within each farm and between farms. Body weights were standardized to 9 weeks of age as the mean age at the second weighing was 59.5 d.

### RNA Extraction

Seventy-four pigs from 4 representative farms with different health status and production parameters were included for gene expression analysis. RNA was extracted from liver tissue using a Qiagen RNeasy Mini Kit (Qiagen). Approximately 30 mg of liver tissue was added to 600 μl of RLT buffer in a RNase-Free tube and homogenized for 30–40 sec using a tissue homogenizer (PowerGen 125, Fisher Scientific). RNA was then extracted from the homogenate as outlined by the manufacturer’s protocol without any modifications (Qiagen). The concentration of the eluted RNA was determined using the Thermo Scientific NanoDrop 8000 Spectrophotometer (Fisher Scientific), and RNA was re-extracted from samples with signs of degradation or contamination.

All RNA samples were treated with an Amplification Grade DNase I kit (Sigma-Aldrich) to remove any genomic DNA. 24 μl of nucleic acid-free water, 3 μl of reaction buffer, 3 μl of Amplification Grade DNase I, and 3 μg of total RNA (1 unit/μl) were added together and centrifuged for 10 seconds to collect the reaction at the bottom of the tube. The mixtures were incubated at room temperature for 15 min. 3 μl of the Stop Solution (50 mM EDTA) was added and the mixtures were incubated at 70°C for 10 min. The mixtures were then chilled on ice before being stored at -80°C.

The purity and concentration of RNA was reassessed after DNase digestion using the Thermo Scientific NanoDrop 8000 Spectrophotometer (Fisher Scientific). The integrity of all the RNA samples was assessed using the Agilent 2100 Bioanalyzer (Agilent Technologies) before and after DNase treatment to ensure there was no indication of degradation. The mean RNA Integrity Number (RIN) was 8.5, and any samples with RIN < 8.0 were repeated as recommended by Fleige and Pfaffl [16].

### Primer design and efficiency

Primer designs and efficiencies are listed in Table 1. Primers for IL-1β, IL-10, IFN-α, and IFN-γ were previously designed by Collado-Romero et al. [17]. All other primer sequences were designed and screened for homology using the NCBI Primer-BLAST program. The cDNA sequences used to design the primers were obtained from the GenBank database. Primers were designed to span known exon-exon junctions. Primer efficiency and the coefficient of determination (R^2^) were determined using the StepOnePlus™ Real-Time PCR System (Applied Biosystems).

**Table 1:** Primers used for RT-qPCR of hepatic biomarkers

**Gene symbol**	**Gene name**	**Accession number**^**a**^	**Primers (5′ – 3′)**	**T**_**m **_**(°C)**	**Amplicon length (bp)**	**R**^**2**^	**Primer efficiency (%)**
*IL1B*	Interleukin-1β	NM_214055	F: GGCCGCCAAGATATAACTGA^b^	59	70	0.991	100
			R: GGACCTCTGGGTATGGCTTTC^b^				
*IL6*	Interleukin-6	NM_214399	F: CCCTGAGGCAAAAGGGAAAGA	60	212	0.998	95.2
			R: CGTGGACGGCATCAATCTCA				
*IL10*	Interleukin-10	L20001	F: CAGATGGGCGACTTGTTG^b^	57	219	0.993	91.7
			R: ACAGGGCAGAAATTGATGAC^b^				
*IL18*	Interleukin-18	NM_213997	F: GCTGCTGAACCGGAAGACAA	60	192	0.996	95.3
			R: AAACACGGCTTGATGTCCCT				
*IFNA*	Interferon-α	AB257591	F: GACCTGCCTCAGATCCACAG	60	158	0.986	82.3
			R: ATGGCTTGAGCCTTCTGGAC				
*IFNG*	Interferon-γ	X53085	F: CAAAGCCATCAGTGAACTCATGA^b^	60	100	0.985	98.7
			R: TCTCTGGCCTTGGAACATAGTCT^b^				
*TNFA*	Tumor necrosis factor-α	NM_214022	F: CCTCTTCTCCTTCCTCCTG^b^	57	194	0.997	100
			R: CCTCGGCTTTGACATTGG^b^				
*CRP*	C-reactive protein	NM_213844	F: TGCCCAGACAGACATGATCG	60	131	0.999	100
			R: GGTCGGTATAGACACGCAGG				
*HP*	Haptoglobin	NM_214000	F: TGAATGTGAAGCAGTGTGCG	59	133	0.996	96
			R: CGAGGTGAGGTTATGGTGGG				
*SAA*	Serum amyloid A	EF362780	F: TGATCAGCGATGCCAGAGAG	60	85	0.998	87.8
			R: CTTGAGTCCTCCACTCCGTG				
*IGF1*	Insulin-like growth factor-1	JX827417	F: TCTTCTACTTGGCCCTGTGCTT	61	81	0.998	97.7
			R: CCAGCTCAGCCCCACAGA				
*IGFBP3*	Insulin-like growth factor binding protein-3	NM_001005156	F: GGCATCCACATCCCCAACT	60	80	0.990	97.3
			R: CCCCGCTTCCTGCCTTT				
*GHR*	Growth hormone receptor	JF276446	F: CTCCACAGGGCCTCGTACTC	60	80	0.999	89.2
			R: GCTCACATAGCCACACGATGA				
*GAPDH*	Glyceraldehyde 3-phosphate dehydrogenase	NM_001206359	F: ACACACCGAGCATCTCCTGACT	61	80	0.998	100
			R: CGAGGCAGGTCTCCCTAAGC				

^a^Accessed via GenBank.

^b^Designed by Collado-Romero et al. [17].

### Analysis of gene expression with RT-qPCR

Relative gene expression was assessed using a real time quantitative polymerase chain reaction (RT-qPCR) assay. The synthesis of cDNA and the RT-qPCR assay were completed by the Advanced Analysis Centre’s Genomic Facility at the University of Guelph. For synthesis of cDNA, 1000 ng of total RNA was reverse transcribed into cDNA using the High Capacity cDNA Reverse Transcription kit (Applied Biosystems). A total reaction volume of 20 μl was used for reverse transcription; 2 μl of reverse transcript buffer, 0.8 μl (100 mM) dNTP, 2 μl random priming oligonucleotides , 1 μl of MultiScribe™ Reverse Transcriptase (50 U/μl) (Applied Biosystems), 4.2 μl of nucleic acid-free water, and 10 μl (1000 ng) of total RNA. The reverse transcription assay was run at 25°C for 5 min, 37°C for 120 min, 85°C for 5 min, and cooled at 4°C.

GAPDH and RLP4 were evaluated as potential reference genes for the present study based on validation and reliable performance in previous research [10,18-20]. The stability of these reference genes in the liver tissue samples was measured using RT-qPCR and analyzed using BestKeeper software [21]. The RT-qPCR cycle quantification (C_q_) values of GAPDH and RLP4 had standard deviations of 0.34 and 0.49, respectively. GAPDH was chosen as the only endogenous reference gene to be used for internal normalization of gene expression data in this study due to its very low variability (C_q_ = 18.17 ± 0.34) for the present samples and its reliable performance as a sole reference gene in previous studies [10,20].

A single StepOnePlus™ Real-Time PCR System (Applied Biosystems) was used for all RT-qPCR assays in this study. The RT-qPCR assay was completed using duplicates of each sample. Each 20 μl reaction consisted of 7.5 μl of 2× PerfeCta SYBR Green FastMix ROX (Quanta BioScience), 5 μl of template cDNA, 1.9 μl of molecular grade water, and 0.6 μl of primer. The final primer concentration in each reaction was 200 nM. The RT-qPCR assay conditions were 95°C for 30 s followed by 40 cycles of 95°C for 15 s and 60°C for 30 s. A melting curve was performed to confirm specificity of the amplicon.

### Statistical analysis

Linear regression methods were used to model production parameters and gene expression (ΔC_q_) in relation to growth performance. First, the C_q_ values of each biomarker were normalized using the C_q_ values of the endogenous reference gene GAPDH as described by the following equation:

(1) $$\Delta C_{q}=\left[C_{q}\text{of}\;\text{target}\;\text{gene}\right]–\left[C_{q}\text{of}\;\text{reference}\;\mathrm{gene}\right]$$

The normalized ΔC_q_ values already exist as the log transformed product of the expression ratios that are conventionally calculated using the 2^-ΔΔCt^ method [22], thereby fulfilling the assumption of linearity. The linearity of the ΔC_q_ data was confirmed by assessing a smoothed locally weighted scatterplot. The ΔC_q_ values were modelled as the magnitude of gene expression for each target gene.

One multivariable random-effect generalized least squares (GLS) model and two multivariable fixed-effect least squares dummy variable (LSDV) models were created in STATA 10.0 (Stata Corporation, College Station, TX). The GLS model was designed to evaluate the association of production characteristics with 9-wk BW across the 8 farms. The two LSDV models were designed to determine the association of biomarker expression with ADG and 9-wk BW across 4 farms. Extraneous variables that were used for model building include the farm, parity of the sow, stillbirths per litter, live births per litter, sex of the piglet, age at weaning, weight at weaning, and disease status. Each model was manually designed using a parsimonious approach. All variables were first screened individually with univariable linear regression. Variables with a liberal p-value of ≤0.20 were included in the initial model. All variables regardless of inclusion were evaluated for confounding. A variable was determined to be a confounder if its inclusion in the model changed the coefficient of any predictor by ≥20%. Two-way interactions were generated for all variables in the initial model and interactions were retained in the model if they were statistically significant (*P* < 0.05). Any variable from the initial model was only retained in the final model if it was statistically significant (*P* < 0.05), if the variable behaved as a confounder, or if the variable was a component of a statistically significant interaction term. If two independent variables presented colinearity (R^2^ > 0.8) then the less significant variable was excluded. Clustering at the farm level was accounted for by modelling each farm as a random-effect in the GLS model and as a fixed-effect in the LSDV models. Homoskedasticity and normal distribution of standardized residuals was assessed graphically for the GLS model. The assumptions of constant variance and normal distribution of standardized residuals was evaluated for each LSDV model using the Cook-Weisberg test and the Shapiro-Wilk test, respectively. Diagnostic analysis revealed no outliers or extremely influential observations. However, observations for two pigs were excluded from the model due to missing data. Effects with a *P* < 0.05 were considered statistically significant and non-significant effects with a *P* < 0.10 were considered to be suggestive of an association. All descriptive statistics are reported as the mean ± standard deviation.

## Results

The mean performance parameters for each farm are presented in Table 2. The average weight at 5-wk post-weaning (59.5 ± 5.5 d) was 19.6 ± 4.3 kg. From weaning to 5-wk post-weaning, a total mortality and culling rate of 2.4% (4/168) was observed. Table 3 summarizes the sow characteristics of each farm.

**Table 2:** Description of piglet growth performance by farm

**Farm**	**Age at weaning (days)**	**Weight at weaning (kg)**	**9-wk BW (kg)**	**ADG (g/day)**
1^a^	26.7 ± 1.19	6.19 ± 1.54	21.9 ± 3.24	399 ± 62.4
2^a^	22.7 ± 3.36	6.41 ± 1.70	17.4 ± 4.31	267 ± 77.0
3^a^	34.4 ± 7.16	8.93 ± 2.00	21.0 ± 3.47	334 ± 72.4
4	28.3 ± 1.06	6.71 ± 1.88	22.2 ± 4.52	306 ± 65.0
5	30.9 ± 2.92	7.95 ± 1.81	20.2 ± 4.32	360 ± 97.1
6	18.7 ± 0.49	7.04 ± 1.51	22.6 ± 3.01	340 ± 47.5
7	26.1 ± 0.74	8.19 ± 1.82	23.0 ± 4.70	387 ± 143.9
8^a^	22.4 ± 1.96	5.81 ± 1.30	17.9 ± 3.20	296 ± 55.5
**Mean**	**26.4 ± 5.59**	**7.16 ± 1.96**	**20.7 ± 4.31**	**336 ± 83.2**

*BW* body weightm, *ADG* post-weaning average daily gain.

^a^Gene expression analysis was performed on liver tissue from the piglets originating from these farms.

**Table 3:** Description of sow and litter characteristics by farm

**Farm**	**Number of sows**	**Sow parity**^**a**^	**Live births per litter**^**a**^	**Still births per litter**^**a**^
1	650	2.3 ± 1.9	12.6 ± 2.8	0.9 ± 0.6
2	500	3.6 ± 2.3	11.4 ± 2.8	1.1 ± 1.0
3	1000	2.3 ± 3.2	13.9 ± 1.3	0.4 ± 1.4
4	240	2.3 ± 1.2	11.0 ± 0.5	0.6 ± 0.5
5	123	4.9 ± 2.2	12.3 ± 2.0	1.4 ± 1.0
6	650	3.1 ± 1.9	13.4 ± 1.7	1.0 ± 1.1
7	850	3.6 ± 2.2	11.3 ± 2.7	1.0 ± 0.8
8	670	4.1 ± 2.9	12.9 ± 3.2	0.1 ± 0.4
**Mean**	**585**	**3.3 ± 2.43**	**12.3 ± 2.46**	**0.8 ± 1.0**

^a^based on the seven randomly selected sows per farm used in this study.

In the first model, sow parity, live births per litter, stillbirths per litter, sex, age at weaning, and the weight at weaning were used as the independent variables to determine an association with 9-wk BW. There were no additional confounding effects or significant interaction terms detected. The final model revealed that only the weight at weaning and the age at weaning were significantly associated with 9-wk BW (Table 4). This model explained 55.8% of the variation in 9-wk BW within each herd. The intraclass correlation coefficient (ICC) was 0.668 based on the herd-level and animal-level variance for the 8 farms.

**Table 4:** Characteristics associated with the weight of pigs at 9 weeks of age (model 1)

**Variable**	**Coefficient**^**a**^	**SE**	**95% CI**	***P *** **value**
Weight at weaning	1.670	0.128	1.419, 1.920	<0.001
Age at weaning	-0.226	0.069	-0.361, -0.090	0.001
Sex	0.524	0.424	-0.307, 1.356	0.217
Parity of sow	0.072	0.097	-0.117, 0.262	0.455
Stillbirths per litter	0.290	0.248	-0.197, 0.777	0.243
Live births per litter	-0.102	0.093	-0.285, 0.081	0.274

*SE* standard error, *CI* confidence interval.

^a^The predicted change in body weight (kg) at 9 weeks of age if the corresponding variable is increased by one unit (ie. the model predicts a decrease of 0.226 kg in body weight at 9 weeks of age if a pig is weaned one day later).

Multivariable analysis revealed ADG was not significantly associated with hepatic gene expression of CRP, SAA, Hp, IFN-α, IFN-γ, TNF-α, IL-1β, IL-6, IL-10, and IL-18 in the liver (*P* > 0.05), and none of these variables were retained in the final model. The effects of IGFBP-3 expression (*P* = 0.016) and GHR expression (*P* = 0.009) on ADG were statistically significant and the effect of IGF-1 expression (*P* = 0.071) was suggestive (Table 5). The model was able to explain 52.9% of the variation in post-weaning ADG. The weight at weaning was retained in the model because it had a confounding effect. All two-way interactions were not significant and thus excluded from the final model.

**Table 5:** Predicted change in ADG given a 2-fold increase in gene expression (model 2)

**Gene**	**Change in ADG (g)**	**SE**	**95% CI**	***P *** **value**
IGF-1	12.85	6.990	-1.11, 26.82	0.071
IGFBP-3	-33.75	13.65	-61.03, –6.48	0.016
GHR	41.76	15.57	10.65, 72.87	0.009

*ADG* post-weaning average daily gain, *SE* standard error, *CI* confidence interval.

Expression of CRP, SAA, Hp, IFN-α, IFN-γ, TNF-α, IL-1β, IL-6, IL-10, and IL-18 in the liver did not affect 9-wk BW (*P* > 0.05), and all of these variables were excluded from the final model. However, the effects of IGFBP-3 expression (*P =* 0.012) and GHR expression (0.016) were significantly associated with 9-wk BW. The effect of IGF-1 expression (*P =* 0.055) was suggestive of an association (Table 6). The model was able to explain 69.4% of the variation in the 9-wk BW. The weaning age and the weight at weaning were retained in the model because both variables had a confounding effect. All two-way interactions were excluded from the model as none were statistically significant.

**Table 6:** Predicted change in 9-wk BW given a 2-fold increase in gene expression (model 3)

**Gene**	**Change in 9-wk BW (kg)**	**SE**	**95% CI**	***P *** **value**
IGF-1	0.547	0.280	-0.012, 1.105	0.055
IGFBP-3	-1.409	0.545	-2.497, –0.320	0.012
GHR	1.580	0.638	0.305, 2.854	0.016

*BW* body weight, *SE* standard error, *CI* confidence interval.

## Discussion

The weight at weaning and the age at weaning both contributed significantly to growth performance in nursery pigs. The weight at weaning was positively associated with body weight during the nursery period which is consistent with results from previous studies [23-25]. Additional research has demonstrated that pigs with higher weaning weights reach market weight 9–15 days earlier [23,26]. These results support the importance of pre-weaning interventions that improve weight at weaning and subsequent performance in the nursery.

Growth performance was negatively associated with age at weaning which implies that pigs weaned at an earlier age will reach a greater body weight during the nursery stage. This finding is in agreement with previous research which indicates that late weaning is disadvantageous for post-weaning growth performance [24]. Research by Main et al. determined that weaning at 21.5 d is optimal for nursery performance in multisite production systems [27]. In the present study, 80.4% of the piglets were >21.5 d old at weaning which is indicative of the negative association observed between weaning age and post-weaning growth performance.

The sex of the piglet was not associated with growth performance in the nursery. These findings consistently agree with previous research [23,24]. Growth performance of gilts and barrows appears to be significantly different only during the grower-finisher phase of production [8,23]. Paredes et al. demonstrated that sex can be significantly associated with nursery performance in different data sets although the authors attributed this consequence to differences in the proportion of intact males [25]. In the present study, all male piglets had been castrated prior to weaning.

Litter characteristics were not associated with nursery growth performance. The parity of the sow, stillbirths per litter, and live births per litter showed no effect on 9-wk BW. There are conflicting reports of the effects of sow parity on post-weaning growth performance. A study by Smith et al. suggests that piglets born to primiparous sows have a growth disadvantage compared to higher parity sows [28]. However, the authors noted that the mean post-weaning weights varied greatly among parties and there was no discernible pattern of growth performance. Other researchers have concluded that sow party does not influence growth performance at the nursery stage [25]. In addition, previous findings have demonstrated that the number of live births per litter is not associated with post-weaning growth performance [25], although the number of live births does appear to be associated with birth weight [29]. One reason for lack of association between sow characteristics and growth performance in this study may have resulted from selecting three piglets of small, medium, and large size from each sow.

Increased expression of IGF-1 in the liver showed a tendency to be positively associated with ADG and 9-wk BW. This is consistent with other studies which have determined that serum IGF-1 concentration is positively associated with growth in pigs and other animals [8,30]. Furthermore, over-expression of IGF-1 at 1.5-fold the normal levels in transgenic mice resulted in a 30% increase in body weight [4]. In this study, a smaller effect was observed in pigs with the model predicting that a 2-fold increase in IGF-1 would result in a 2.6% increase in body weight at 9 weeks of age for the average nursery pig. The statistical significance of the association between IGF-1 and growth may have been undermined by the large variation in IGF-1 RNA levels. The variation in hepatic IGF-1 expression is likely due to the natural variability observed during this stage of development [1,30]. Additionally, differences in production management, feed composition, and antimicrobial usage may have contributed to the large variation in hepatic IGF-1 expression [31]. It is likely that differences in breed and sex across farms were not a factor in variation as previous studies have demonstrated that IGF-1 levels are not significantly different between breeds [10,32] or between barrows and gilts [30]. Expression of IGF-1 is likely more useful as a growth-associated biomarker for comparing pigs that are raised in the same herd under the same conditions.

IGFBP-3 expression in the liver was negatively associated with ADG and 9-wk BW. The model designed in this experiment predicted that a 2-fold increase in IGFBP-3 expression would reduce 9-wk BW by 6.8% in the average nursery pig. Contrary to the results of our findings, previous research by Owens et al. showed a weak positive correlation between serum IGFBP-3 concentration and ADG [30]. However, other research has been demonstrated that IGFBP-3 is expressed significantly less in gilts bred for enhanced growth performance compared to gilts with diminished growth performance [8], and that hepatic expression of IGFBP-3 is higher in slow-growing breeds than in fast-growing breeds of pigs [10]. Further research using transgenic mice found that a 4.9-7.7-fold increase in IGFBP-3 expression resulted in a 10% reduction in birth weight, moderate post-natal growth retardation, and reduced organ weight [7]. Over-expression of IGFBP-3 in transgenic mice also resulted in a 1.9-2.8-fold increase in circulating IGF-1 levels [7], a finding that can be explained by the concomitant mechanisms that regulate both IGF-1 and IGFBP-3 expression [33,34]. It is likely that neglecting to account for IGF-1 levels during analysis lead Owens et al. to discover a weak positive association between IGFBP-3 and growth performance [30]. Re-modelling the data from our study without IGF-1 confirmed that IGF-1 does have a major confounding effect on the IGFBP-3 coefficient. Overall, the significant negative association between IGFBP-3 and growth performance found in the present study is consistent with findings in other studies [7,8,10].

Expression of GHR in the liver was positively associated with growth performance in nursery pigs. A 2-fold increase in GHR expression appeared to have the greatest effect on the magnitude of growth compared to the same increase in IGF-1 and IGFBP-3 expression. A transgenic experiment with mice demonstrated that deletion of the GHR gene resulted in decreased circulating IGF-1 and a substantial reduction in body weight [35]. These findings are consistent with the results presented in this paper. Further studies have demonstrated that in *vitro* GHR expression of swine hepatocytes is significantly influenced by glucose and amino acid concentrations [36], and *in vivo* experiments with pigs indicate that dietary protein concentration is positively associated with GHR expression in the liver, skeletal muscle, and adipose tissue [37]. In light of the results from the present study, assessing expression of GHR may be useful for evaluating the effects of different dietary regiments on growth performance at the molecular level.

ADG and 9-wk BW were not associated with hepatic expression of the APPs assessed in this study. A previous experiment by Saco et al. demonstrated that Hp is a suitable biomarker for assessing the effects of an immunomodulating feed additive on production parameters [15]. However, Saco et al. were unable to exclusively identify an association between Hp and growth performance [15]. Additional research by Pineiro et al. demonstrated differential serum concentrations of CRP and Hp between two groups of pigs subjected to different feeding regiments [14]. Although the ADG was significantly different between groups, Pineiro et al. attributed the differences in APP levels to stress caused by disorderly feeding [14]. In this study it is likely that no significant association was found between APP expression and growth performance because APP levels are influenced considerably by pathogen colonization and stress [14,38]; two factors which are not necessarily indicative of growth performance. The results of the present study indicate that CRP, Hp, and SAA are not suitable biomarkers for evaluating the production performance of nursery pigs on commercial farms.

The expression of IFN-α, IFN-γ, TNF-α, IL-1β, IL-6, IL-10, and IL-18 in the liver was not significantly associated with growth performance in nursery pigs. Earlier studies have demonstrated that body weight is significantly associated with circulating levels of IL-6, IL-8, IL-18, and TNF-α in humans [39,40], and differential expression of IL-1, IL-6, and TNF-α in horses [41]. The lack of association between body weight and hepatic cytokine expression observed in this study is likely due to previous findings which indicate that excess adipose tissue is the primary source of increased levels of circulating cytokines [42-44]. Monitoring adipose-associated cytokines may be useful for evaluating obesity in other species. Overall, cytokine expression in the liver is unsatisfactory for monitoring growth parameters in nursery pigs, although adipose or serum levels of these cytokines may still prove to be useful biomarkers to evaluate growth performance.

One of the limitations of this study was the lack of dietary information for the swine herds. However, diet is uniform on each farm and farm-level clustering was accounted for in each regression model during the statistical analysis. Furthermore, it may be argued that diet acts as an intervening variable which can introduce considerable variation into the data but would not need to be controlled for the results to be valid.

Measuring the hepatic expression of IGF-1, IGFBP-3, and GHR can be useful biomarkers for evaluating the growth performance of nursery pigs. It is likely that serological levels of IGF-1, IGFBP-3, and GHR are representative of the changes in liver expression levels, however further research is needed to confirm this assumption. The advantages of evaluating gene expression is that it allows for the determination of growth-associated biological activity in specific tissues and it demonstrates that transcriptional diversity is related to overall growth performance. Hence, the results of this study should direct future research to explore whether any polymorphisms in growth factor genes (*IGF1*, *IGFBP3*, *GHR),* or accessory genes such as proteases, are associated with transcriptional diversity and indicative of growth performance in pigs. Identifying polymorphisms related to growth factor expression may improve the selection of genetic lineages for enhanced production performance. Quantifying IGF-1, IGFBP-3, and GHR may also be beneficial in research and experimental settings where they may be used, in addition to conventional methods, to assess interventions, treatments, or clinical pathologies in relation to growth performance of nursery pigs. Future research should focus on whether RNA and serum levels of IGF-1, IGFBP-3, and GHR are indicative of growth performance at other phases of swine production and whether biomarker expression during the nursery phase can predict performance in later phases of production.

## Conclusion

Overall, IGFBP-3 and GHR expression in the liver was significantly associated with growth performance across multiple swine production systems despite variation in operating procedures. IGF-1 expression had a tendency to be associated with the measured growth parameters but the large variation reduced its statistical significance in this study. Nevertheless, IGF-1 is likely a useful biomarker of growth performance for comparing pigs from the same herd. IGFBP-3 and GHR appear to be suitable biomarkers for evaluating growth performance in nursery pigs, either in a controlled experimental setting or in population-based studies involving multiple swine herds with varying management practices. Hepatic expression of CRP, Hp, SAA, IFN-α, IFN-γ, TNF-α, IL-1β, IL-6, IL-10, and IL-18 was not associated with the growth parameters in this study and appear to be inadequate for monitoring growth in pigs.

## Competing interests

The authors of this research declare that they have no competing interests.

## Authors’ contributions

MJS: Field and post-mortem sample collection; RNA extraction, quantification, and qualification; coordinated RT-qPCR; data analysis; writing manuscript. RF: Recruitment of swine producers; coordinated field work; interpretation of data analysis; critical review. CFMDL: Study design and oversight; coordinated RT-qPCR; interpretation of results. MR: post-mortem sample collection; primer design; RNA extraction; DNase treatment; interpretation of results. The final manuscript was read and approved by all authors. AF: Designed study and survey; recruitment of swine producers; pre-mortem examinations and autopsies; data analysis. All authors read and approved the final manuscript.

## References

[B1] Dupont J Holzenberger M Biology of insulin-like growth factors in development Birth Defects Res C Embryo Today 2003 9 4 257 271 10.1002/bdrc.10022 14745968

[B2] Florini JR Ewton DZ Coolican SA Growth hormone and the insulin-like growth factor system in myogenesis Endocr Rev 1996 9 5 481 517 8897022 10.1210/edrv-17-5-481

[B3] Bichell DP Kikuchi K Rotwein P Growth hormone rapidly activates insulin-like growth factor I gene transcription in vivo Mol Endocrinol 1992 9 11 1899 1908 10.1210/me.6.11.1899 1480177

[B4] Mathews LS Hammer RE Behringer RR D’Ercole AJ Bell GI Brinster RL Palmiter RD Growth enhancement of transgenic mice expressing human insulin-like growth factor I Endocrinology 1988 9 6 2827 2833 10.1210/endo-123-6-2827 3197646

[B5] Mohan S Baylink DJ IGF-binding proteins are multifunctional and act via IGF-dependent and -independent mechanisms J Endocrinol 2002 9 1 19 31 10.1677/joe.0.1750019 12379487

[B6] Rajaram S Baylink DJ Mohan S Insulin-like growth factor-binding proteins in serum and other biological fluids: regulation and functions Endocr Rev 1997 9 6 801 831 10.1210/er.18.6.801 9408744

[B7] Modric T Silha JV Shi Z Gui Y Suwanichkul A Durham SK Powell DR Murphy LJ Phenotypic manifestations of insulin-like growth factor-binding protein-3 overexpression in transgenic mice Endocrinology 2001 9 5 1958 1967 10.1210/en.142.5.1958 11316761

[B8] Clutter AC Spicer LJ Woltmann MD Grimes RW Hammond JM Buchanan DS Plasma growth hormone, insulin-like growth factor I, and insulin-like growth factor binding proteins in pigs with divergent genetic merit for postweaning average daily gain J Anim Sci 1995 9 1776 1783 7545660 10.2527/1995.7361776x

[B9] Wang L Zhang G Lin F Jiang B Dong F Liu H Expression of the insulin-like growth factor system in skeletal muscle during embryonic and postnatal development in the first filial generation pigs from Erhualian and Yorkshire reciprocal crosses Gen Comp Endocrinol 2011 9 1 56 62 10.1016/j.ygcen.2011.04.025 21570979

[B10] Li Z Wu Z Ren G Zhao Y Liu D Expression patterns of insulin-like growth factor system members and their correlations with growth and carcass traits in Landrace and Lantang pigs during postnatal development Mol Biol Rep 2013 9 5 3569 3576 10.1007/s11033-012-2430-1 23269622

[B11] Pierzchała M Pareek CS Urbański P Goluch D Kamyczek M Różycki M Smoczynski R Horbańczuk JO Kurył J Study of the differential transcription in liver of growth hormone receptor (GHR), insulin-like growth factors (IGF1, IGF2) and insulin-like growth factor receptor (IGF1R) genes at different postnatal developmental ages in pig breeds Mol Biol Rep 2012 9 3 3055 3066 10.1007/s11033-011-1068-8 21695430

[B12] Eurell TE Bane DP Hall WF Schaeffer DJ Serum haptoglobin concentration as an indicator of weight gain in pigs Can J Vet Res 1992 9 1 6 9 1586895 PMC1263495

[B13] Stevenson LS McCullough K Vincent I Gilpin DF Summerfield A Nielsen J McNeilly F Adair BM Allan GM Cytokine and C-reactive protein profiles induced by porcine circovirus type 2 experimental infection in 3-week-old piglets Viral Immunol 2006 9 2 189 195 10.1089/vim.2006.19.189 16817761

[B14] Piñeiro C Piñeiro M Morales J Carpintero R Campbell FM Eckersall PD Toussaint MJ Alava MA Lampreave F Pig acute-phase protein levels after stress induced by changes in the pattern of food administration Animal 2007 9 1 133 139 10.1017/S1751731107283909 22444216

[B15] Saco Y Fraile L Giménez M Pato R Montoya M Bassols A Haptoglobin serum concentration is a suitable biomarker to assess the efficacy of a feed additive in pigs Animal 2010 9 9 1561 1567 10.1017/S1751731109991418 22444704

[B16] Fleige S Pfaffl MW RNA integrity and the effect on the real-time qRT-PCR performance Mol Aspects Med 2006 9 2–3 126 139 16469371 10.1016/j.mam.2005.12.003

[B17] Collado-Romero M Arce C Ramírez-Boo M Carvajal A Garrido JJ Quantitative analysis of the immune response upon *Salmonella typhimurium* infection along the porcine intestinal gut Vet Res 2010 9 2 23 10.1051/vetres/2009072 19941811 PMC2820228

[B18] Skovgaard K Mortensen S Poulsen KT Angen Ø Heegaard PM Validation of putative reference genes for qRT-PCR normalization in tissues and blood from pigs infected with Actinobacillus pleuropneumoniae Vet Immunol Immunopathol 2007 9 1–2 140 146 17544155 10.1016/j.vetimm.2007.04.010

[B19] Svobodová K Bílek K Knoll A Verification of reference genes for relative quantification of gene expression by real-time reverse transcription PCR in the pig J Appl Genet 2008 9 3 263 265 10.1007/BF03195623 18670063

[B20] Li Z Cao B Zhao B Yang X Fan MZ Yang J Decreased expression of calpain and calpastatin mRNA during development is highly correlated with muscle protein accumulation in neonatal pigs Comp Biochem Physiol A Mol Integr Physiol 2009 9 4 498 503 10.1016/j.cbpa.2008.12.004 19130893

[B21] Pfaffl MW Tichopad A Prgomet C Neuvians TP Determination of stable housekeeping genes, differentially regulated target genes and sample integrity: BestKeeper–Excel-based tool using pair-wise correlations Biotechnol Lett 2004 9 6 509 515 15127793 10.1023/b:bile.0000019559.84305.47

[B22] Livak KJ Schmittgen TD Analysis of relative gene expression data using real-time quantitative PCR and the 2(-Delta Delta C(T)) Method Methods 2001 9 4 402 408 10.1006/meth.2001.1262 11846609

[B23] Wolter BF Ellis M The effects of weaning weight and rate of growth immediately after weaning on subsequent pig growth performance and carcass characteristics Can J Anim Sci 2001 9 3 363 369 10.4141/A00-100

[B24] De Grau A Dewey C Friendship R De Lange K Observational study of factors associated with nursery pig performance Can J Vet Res 2005 9 4 241 245 16479720 PMC1250234

[B25] Paredes SP Jansman AJ Verstegen MW Awati A Buist W Den Hartog LA Van Hees HM Quiniou N Hendriks WH Gerrits WJ Analysis of factors to predict piglet body weight at the end of the nursery phase J Anim Sci 2012 9 9 3243 3251 10.2527/jas.2011-4574 22585823

[B26] Mahan DC Lepine AJ Effect of pig weaning weight and associated nursery feeding programs on subsequent performance to 105 kilograms body weight J Anim Sci 1991 9 4 1370 1378 2071501 10.2527/1991.6941370x

[B27] Main RG Dritz SS Tokach MD Goodband RD Nelssen JL Increasing weaning age improves pig performance in a multisite production system J Anim Sci 2004 9 5 1499 1507 15144093 10.2527/2004.8251499x

[B28] Smith AL Stalder KJ Serenius TV Baas TJ Mabry JW Effect of piglet birth weight on weights at weaning and 42 days post weaning J Swine Health Prod 2007 9 4 213 218

[B29] Beaulieu AD Aalhus JL Williams NH Patience JF Impact of piglet birth weight, birth order, and litter size on subsequent growth performance, carcass quality, muscle composition, and eating quality of pork J Anim Sci 2010 9 8 2767 2778 10.2527/jas.2009-2222 20418451

[B30] Owens PC Gatford KL Walton PE Morley W Campbell RG The relationship between endogenous insulin-like growth factors and growth in pigs J Anim Sci 1999 9 8 2098 2103 10461987 10.2527/1999.7782098x

[B31] Hathaway MR Dayton WR White ME Henderson TL Henningson TB Serum insulin-like growth factor I (IGF-I) concentrations are increased in pigs fed antimicrobials J Anim Sci 1996 9 7 1541 1547 8818798 10.2527/1996.7471541x

[B32] Brameld JM Atkinson JL Budd TJ Saunders C Pell JM Salter AM Gilmour RS Buttery PJ Expression of insulin-like growth factor-1 (IGF-1) and growth hormonereceptor (GHR) mRNA in liver, skeletal muscle and adipose tissue of different breeds of pig Anim Sci 1996 9 3 555 559 10.1017/S1357729800015101

[B33] Ramajayam G Vignesh RC Karthikeyan S Kumar KS Karthikeyan GD Veni S Sridhar M Arunakaran J Aruldhas MM Srinivasan N Regulation of insulin-like growth factors and their binding proteins by thyroid stimulating hormone in human osteoblast-like (SaOS2) cells Mol Cell Biochem 2012 9 1–2 77 88 22673962 10.1007/s11010-012-1345-4

[B34] Vottero A Guzzetti C Loche S New aspects of the physiology of the GH-IGF-1 axis Endocr Dev 2013 9 96 105 23392098 10.1159/000342573

[B35] Coschigano KT Holland AN Riders ME List EO Flyvbjerg A Kopchick JJ Deletion, but not antagonism, of the mouse growth hormone receptor results in severely decreased body weights, insulin, and insulin-like growth factor I levels and increased life span Endocrinology 2003 9 9 3799 3810 10.1210/en.2003-0374 12933651

[B36] Brameld JM Gilmour RS Buttery PJ Glucose and amino acids interact with hormones to control expression of insulin-like growth factor-I and growth hormone receptor mRNA in cultured pig hepatocytes J Nutr 1999 9 7 1298 1306 10395590 10.1093/jn/129.7.1298

[B37] Brameld JM Atkinson JL Saunders JC Pell JM Buttery PJ Gilmour RS Effects of growth hormone administration and dietary protein intake on insulin-like growth factor I and growth hormone receptor mRNA Expression in porcine liver, skeletal muscle, and adipose tissue J Anim Sci 1996 9 8 1832 1841 8856437 10.2527/1996.7481832x

[B38] Gómez-Laguna J Salguero FJ Pallarés FJ Fernández De Marco M Barranco I Cerón JJ Martínez-Subiela S Van Reeth K Carrasco L Acute phase response in porcine reproductive and respiratory syndrome virus infection Comp Immunol Microbiol Infect Dis 2010 9 6 e51 e58 10.1016/j.cimid.2009.11.003 20004019

[B39] Esposito K Pontillo A Ciotola M Di Palo C Grella E Nicoletti G Giugliano D Weight loss reduces interleukin-18 levels in obese women J Clin Endocrinol Metab 2002 9 8 3864 3866 10.1210/jc.87.8.3864 12161523

[B40] Bruun JM Verdich C Toubro S Astrup A Richelsen B Association between measures of insulin sensitivity and circulating levels of interleukin-8, interleukin-6 and tumor necrosis factor-alpha: effect of weight loss in obese men Eur J Endocrinol 2003 9 5 535 542 10.1530/eje.0.1480535 12720537

[B41] Vick MM Adams AA Murphy BA Sessions DR Horohov DW Cook RF Shelton BJ Fitzgerald BP Relationships among inflammatory cytokines, obesity, and insulin sensitivity in the horse J Anim Sci 2007 9 5 1144 1155 10.2527/jas.2006-673 17264235

[B42] Bastard JP Jardel C Bruckert E Blondy P Capeau J Laville M Vidal H Hainque B Elevated levels of interleukin 6 are reduced in serum and subcutaneous adipose tissue of obese women after weight loss J Clin Endocrinol Metab 2000 9 9 3338 3342 10.1210/jc.85.9.3338 10999830

[B43] Coppack SW Pro-inflammatory cytokines and adipose tissue Proc Nutrition Soc 2001 9 3 349 356 10.1079/PNS2001110 11681809

[B44] Fain JN Release of interleukins and other inflammatory cytokines by human adipose tissue is enhanced in obesity and primarily due to the nonfat cells Vitam Horm 2006 9 443 477 17027526 10.1016/S0083-6729(06)74018-3

